# Genetic Analysis of Sodium Channel Genes in Pediatric Epilepsy Patients of Pakistan

**DOI:** 10.1155/2022/1168703

**Published:** 2022-01-29

**Authors:** Aqsa Ashfaq, Tayyaba Saleem, Nadeem Sheikh, Hafsa Maqbool

**Affiliations:** ^**1**^ Institute of Zoology, University of the Punjab Lahore, Lahore, Pakistan; ^2^Department of Neurology, University Medical Center Göttingen, Robert-Koch-Straße 40, Göttingen 37075, Germany

## Abstract

Epilepsy affects millions of people worldwide. Although antiepileptic drugs work for the majority of epileptic patients, these drugs do not work for some of the patients, subjecting them to drug-resistant epilepsy (DRE). Voltage-gated sodium channels act as targets for a number of antiepileptic drugs, and the genes encoding these channels can play a crucial role in developing drug-resistant epilepsy. This case-control (100 control: 101patients) study evaluated the association of sodium channel genes SCN1A and SCN2A with drug-resistant epilepsy. The cases were further accounted in two categories, drug-resistant and drug-responsive epileptic patients. The polymorphic sites rs794726754, rs1057518252, rs121918809, rs12191792, rs121917932, c.730 G > T, c.735 G > T, c.736 A > T, rs10167228, and rs2298771 of the SCN1A gene and rs17183814 of SCN2A gene were selected for mutational analysis. The DNA was isolated, amplified by PCR, and then, was run through 1% agarose gel. The sequencing was performed, and the sequences were observed through BioEdit software for any change in DNA sequence. In our study, no polymorphism was observed in the studied SNPs except for rs2298771. For rs2298771, a significant difference existed in the distribution of genotypic and allelic frequencies (*p* < 0.01) among the case and control group. Furthermore, the genotypic and allelic frequencies of the two categories of cases (drug responder drug resistant) were calculated. The genotypic and allelic frequencies of drug-responsive and drug-resistant epileptic patients did not differ significantly (*p* > 0.01). Our study indicated that the rs2298771 polymorphism of SCN1A may not be associated with chance of developing DRE in the Pakistani population.

## 1. Introduction

Epilepsy is a neurological disorder that is described by intermittent fits and seizures [1]. Epilepsy was characterized theoretically in 2005 as a disorder of the brain described by repeatedly occurring epileptic seizures [2].Several antiepileptic drugs have been developed to treat epileptic seizures, but not all the patients respond to the drugs. Approximately 20% of the epileptic patients do not respond to drugs and are considered to be drug resistant and continue to have debilitating seizures [3].

Drug-resistant epilepsy is defined as consistency of epileptic seizures despite recommended use of antiepileptic drugs [4]. Regardless of the fact that many advances in this field have been made to develop new antiepileptic drugs with improved efficacy, there is no significant decrease in the proportion of patients with drug-resistant epilepsy [5]. The mechanism for the development of drug resistance is not fully understood [6]. Multiple hypotheses were proposed to understand the underlying mechanism of drug resistance. The target and transporter hypothesis are two well-understood hypotheses for explaining drug resistance [7,8].

Voltage-gated ion channels have an important role in triggering epilepsy, and also, they act as targets for a number of antiepileptic drugs [9]. Voltage-gated sodium channels play a crucial role in generating action potential as well as in membrane excitability. Voltage-gated sodium channels consist of *α* and *ß* subunits. Any noticeable change or anomaly in subunits of sodium channels alter their activation; i.e., they are activated at a slower rate. Because of slower activation, the membrane remains depolarized for a longer period of time and can be the cause of epilepsy and generation of epileptic seizures [10,11].

Neuronal voltage-gated sodium channels act as targets for a number of antiepileptic drugs (AEDs), i.e., carbamazepine, phenytoin, and valproate. Aside from their job in nerve conduction and the process of epileptogenesis, these voltage-gated sodium channels are likewise perceived as the significant focuses with respect to AED viability [12,13]. The sodium channel genes SCN1A and SCN2A encode the *α* subunit of voltage-gated sodium channels, and thus, mutations in these genes can be potential source of drug resistance in epilepsy [14,15].

Single-nucleotide polymorphisms (SNPs) are the most common variations in the human genome which cause changes in efficacy, suitability, and duration of drug action, and they can be considered as possible factors for drug-resistant epilepsy [16]. SCN1A and SCN2A are reported to be associated with effectiveness of drug therapy whether it is mono or multiple antiepileptic drug therapy, but the results of different investigations are contradicted. For instance, SCN1A (rs2298771) is found to be significantly associated with response to antiepileptic drugs in epileptic patients [17], while some studies reported that the SCN1Ars2298771is not linked with responsiveness to drugs, and similar is the case with SCN2A [18].

SCN1Ars3812718 G > A SNP is found to be linked with regular and maximum use of antiepileptic drugs carbamazepine and phenytoin [19–21], but there can be population variations as this association was not found to be significant in the Austrian population [22].

Keeping in view the unsure role of SCN1A SNPs, we, for the first time in the Pakistani population, analyzed the association pattern of these SNPs in drug-resistant epilepsy.

## 2. Materials and Methods

### 2.1. Subjects/Participants

The study was conducted on 201 individuals, consisting of 101 patients and 100 controls. The patients were further accounted in two groups: 42 were drug resistant, and 59 were drug responders. All the drug-resistant patients were clinically diagnosed by neurophysiologists. The main diagnostic criterion was continuation of seizures after following at least two recommended and appropriately chosen therapeutic regimens. The average age of drug responders was 5.5 ± 2.2 years, while the average age of drug resistant was 6.5 ± 2.4 years. Controls were healthy individuals having no physical illness or any history of neurological disorder. The mean age of controls was 6.1 ± 2.3 years. All the subjects of this study were of the same ethnic region to exclude potential biases.

This study was ethically approved by the Bioethics Committee of the University of Punjab, Lahore, Pakistan.

### 2.2. Genotyping

Blood samples of all participants were collected in EDTA-coated tubes (Catalogue No.17091619). Genomic DNA was isolated using an organic method [23]. Exon 6(730 G > T, 735G > T, and 736 A > *T*), exon 26(rs794726754, rs1057518252, rs121918809, rs12191792, and rs121917932), rs10167228, and rs2298771 of the SCN1A gene and rs17183814 of SCN2A were selected for mutational analysis. The targeted SNPs were amplified by polymerase chain reaction using reported primers (Table 1). The primers were optimized at 57°C, 59.5°C, 61°C, 58°C, and 58.5°C, respectively. The targeted amplicons were checked by performing gel electrophoresis after amplification by PCR. The cycling conditions of PCR were as follows: denaturation at 95°C for 5 mins followed by denaturation at 95°C for 30 sec, annealing for 45 sec at the optimized temperatures for selected SNP, and extension at 72°C for 45 sec. Sanger's sequencing was performed from a commercial source (Base Asia Singapore) to detect any change in the DNA sequence of the patient and control group.

### 2.3. Genotypic and Statistical Analysis

The sequences were analyzed by performing BLAST and were also visualized through BioEdit software (7.2.0) for detection of any mutation. Association analysis between controls and drug-resistant epilepsy patients for rs2298771 was conducted by applying the chi-square test through SHEsis online (http://analysis.bio-x.cn/myAnalysis.php). The distribution of genotypic and allelic frequencies was checked by Hardy–Weinberg Equilibrium (HWE). Association analysis between drug-responder epileptic patients and drug-resistant epileptic patients was also performed to check the distribution of wild and mutant allele.

### 2.4. In Silico Analysis

**Figure 1:**
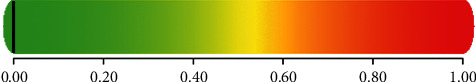
PolyPhen-2 prediction of rs2298771 of SCN1A.

Search Tool for the Retrieval of Interacting Genes (STRING) was used to study protein-protein interaction for the selected genes. PolyPhen-2 software was used to check the pathogenicity of the studied polymorphism. PolyPhen-2 predicts the pathogenicity of a polymorphism based on pathogenicity score. If the pathogenicity score is near 1, the polymorphism is predicted to be damaging, and if the pathogenicity score is near to 0, the polymorphism is predicted to be benign (Figure 1).

## 3. Results

**Figure 2:**
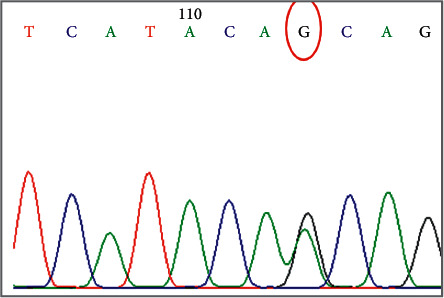
Chromatogram of single-nucleotide changes (*G* > A) in the SCN1A gene (rs2298771).

The clinical data of the subjects are presented in Table 2. In this study, there was no association between SCN2Ars17183814 and DRE. Exon 6, 26, and rs10167228 polymorphisms of SCN1A were also not associated with drug-resistant epilepsy. SCN1Ars2298771 polymorphism was observed in patients of DRE. No significant association was shown between rs2298771 polymorphism and DRE. The distribution of frequencies was also checked by HWE, and it was not violated. The distribution of genotypic and allelic frequencies of cases and controls is given in Table 3. The frequency of GG was higher in controls than in cases, while that of GA was higher in cases. No significant difference was shown in the distribution of genotypic and allelic frequencies between drug-resistant and drug-responsive patients (Table 4). The *p* value for genotypic distribution is 0.14 which showed there is no significant difference between two groups. The results are presented in Table 4. The chromatogram showing mutation is shown in Figure 2.

### 3.1. In Silico Analysis

**Figure 3:**
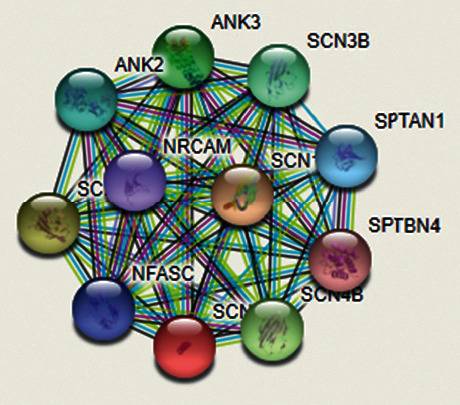
Protein interaction network of SCN1A.

Figure 3 shows the protein network highlighting functional association of SCN1A with other proteins. A node is showing a protein, and the edge is showing predicted functional association. Different line colors show the evidence types for the association. Red lines show the fusion evidence, yellow lines show miming evidence, blue line show database evidence, and black lines show coexpression evidence.

Figure 1 shows the pathogenicity score of rs2298771. According to pathogenicity score by PolyPhen-2 which is 0, r2298771 is likely to be benign.

## 4. Discussion

About twenty percent of epilepsy patients worldwide show resistance to antiepileptic drugs which leads to various psychological problems, i.e., depression and anxiety. Continuous exposure to ineffective drugs can affect the quality of life of drug-resistant patients [28]. Most of the antiepileptic drugs work by affecting sodium channels and blocking them, so the genes encoding these channels are the most obvious candidates for studying genetic polymorphisms affecting drug response [29–31].

Sodium channel genes are the most important targets for antiepileptic drugs affecting their potency. In this study, the association of sodium channel genes SCN1A and SCN2A polymorphisms with drug-resistant epilepsy was studied. The results showed no significant association of SCN1Ars2298771 with DRE, and also, no association was observed between SCN2A and drug resistance.

A significant association of SCN1Ars2298771 with DRE was observed in the north Indian population where the frequency of the AG genotype was significantly higher in patients suffering from drug-resistant epilepsy than in healthy control individuals, but a weak association was observed between SCN2Ars17183814 and drug-resistant epilepsy [14].

In many studies, intronic polymorphisms of SCN1A have also been described to be significantly associated with response to AEDs. Contradictory to our results, a study conducted on 120 epilepsy pediatric patients showed a significant association between intronic SNPs of the SCN1A gene and drug resistance, but no association was observed between exonic SNPs of SCN1A and drug-resistant epilepsy. Three intronic SNPs of SCN1A, rs6730344, rs6732655, and rs10167228, were shown to be potential risk factors for developing drug resistance [3].

In accordance with results of our study, a study conducted on epileptic patients to investigate the effect of valproic acid (VPA), one of the widely used drug for treatment of epilepsy, revealed that SCN1Ars2298771 and SCN2Ars17183814 do not significantly affect response to VPA. Hence, these polymorphisms were not detected to be significantly linked with DRE in the Chinese population [32].

The lack of association of SCN2Ars17183814 with response to antiepileptic drugs in Chinese epileptic patients was also confirmed in [33]. A study conducted on the Scottish population concluded a weak association of SCN2Ars17183814 with DRE [34].

This study showed no mutations in exon 6 of the SCN1A gene. The previous studies have reported mutations in exon 6 of SCN1A which lead to truncation of channel protein [24]. No significant mutation in exon 26 of SCN1A was observed in this study which is consistent with the fact that there are no mutations reported in exon 26 of the SCN1A gene causing drug-resistant epilepsy. However, mutational analysis showed that frameshift mutation in exon 26 of SCN2A can lead to premature stop codon which can affect the protein structure leading to myoclonic epilepsy [25].

## 5. Conclusions

Voltage-gated ion channels have an important role in triggering epilepsy, and also, they act as targets for a number of antiepileptic drugs. The current study has shown that rs2298771 of the SCN1A gene may be involved in the epilepsy susceptibility but not in the occurrence of drug-resistant epilepsy in the Pakistani population. However, more studies from different ethnicities in Pakistan and worldwide are required to evaluate the role of sodium-channel genes in developing drug-resistant epilepsy so that potential targets can be explored for the antiepileptic drugs.

## Figures and Tables

**Table 1:** Primers used for the selected SNPs.

Gene	SNP/exon	Forward primer	Reverse primer	References
SCN1A	EXON 6	AGTGTTGCTTCTCCACTAGC	CGAAGGCACATTAGCAATGA	[24]
	EXON 26	AGGACTCTGAACCTTACCTTGG	TGTACATGTTCACCACAACCAG	[25]
	rs10167228	CCAAATGGTGACACAGTGAA	GCCTTGATCACTTGTAGGACTTTT	[3]
	rs2298771	TGCACAAAGGAGTAGCTTATG	AGTCAAGATCTTTCCCAATTTCTG	[26]
SCN2A	rs17183814	AATCACCTTTTATTCTAATGGTC	CAGTGAAGGCAACTTGACTAAGA	[27]

**Table 2:** Clinical data of the epilepsy patients.

		Drug responders, *N* = 59	Drug resistant, *N* = 42
Age (mean ± S.D)		5.5 ± 2.2	6.5 ± 2.4
Gender	Male	31	25
		28	17
	Female		
Family history	First-degree relatives	29	25
	Second-degree relatives	5	8
Myoclonic jerks		4	0
Tonic seizures		10	0
GTC seizures		30	32
Seizure type: absence seizures		0	2
Atonic		0	4
Infantile spasms		6	2
Juvenile absence seizures		9	2

GTC : generalized tonic clonic.

**Table 3:** Distribution of genotypic and allelic frequencies among cases and controls.

Genotypes/alleles	Case (frequency) (*N* = 101)	Control (frequency) (*N* = 100)	*p* value	OR (95% CI)
GG	0.35	0.59	0.0005^*∗∗*^	2.713(1.532–4.806)
GA	0.65	0.41	0.0005^*∗∗*^	
G	0.67	0.79	0.005^*∗*^	1.881(1.197–2.957)
A	0.33	0.21	0.005^*∗*^	

Data are presented as number (N) and frequency ^*∗*^*p* < 0.01, ^*∗∗*^*p* < 0.001, and ^*∗∗∗*^*p* < 0.0001. OR: odds ratio; CI : confidence interval.

**Table 4:** Distribution of genotypic and allelic frequency between drug-responsive and drug-resistant epileptic patients.

Genotypes/alleles	Patients (frequency)		*p* value	OR (95%CI)
	Drug responders, N = **59**	Drug resistant, N = **42**		
GG	0.29	0.43	0.14	0.539(0.235–1.239)
GA	0.71	0.57	0.14	
G	0.65	0.72	0.29	0.723(0.395–1.325)
A	0.35	0.28	0.29	

Data are presented as number (N) and frequency. OR: odds ratio; CI : confidence interval.

## Data Availability

The data used to support the findings of this study are available upon request from the corresponding author.

## References

[1] Nazish H. R., Ullah N., Ullah S. (2018). The possible effect of SCN1A and SCN2A genetic variants on carbamazepine response among Khyber Pakhtunkhwa epileptic patients, Pakistan. *Therapeutics and Clinical Risk Management*.

[2] Fisher R. S., Acevedo C., Arzimanoglou A. (2014). ILAE official report: a practical clinical definition of epilepsy. *Epilepsia*.

[3] Margari L., Legrottaglie A. R., Vincenti A. (2018). Association between SCN1A gene polymorphisms and drug resistant epilepsy in pediatric patients. *Seizure*.

[4] Kwan P., Arzimanoglou A., Berg A. T. (2010). *Definition of Drug Resistant Epilepsy: Consensus Proposal by the Ad Hoc Task Force of the ILAE Commission on Therapeutic Strategies*.

[5] Chen Z., Brodie M. J., Kwan D., Kwan P. (2018). Treatment outcomes in patients with newly diagnosed epilepsy treated with established and new antiepileptic drugs. *JAMA Neurology*.

[6] Sánchez M. B., Herranz J. L., Leno C. (2010). Genetic factors associated with drug-resistance of epilepsy: relevance of stratification by patient age and aetiology of epilepsy. *Seizure*.

[7] Kasperavičiūtė S. M. (2009). Epilepsy pharmacogenetics. *Pharmacogenetics*.

[8] Rogawski M. A., Johnson M. R. (2008). Intrinsic severity as a determinant of antiepileptic drug refractoriness. *Epilepsy Currents*.

[9] Kumari R., Lakhan R., Garg R., Kalita J., Mittal B. (2011). Pharmacogenomic association study on the role of drug metabolizing, drug transporters and drug target gene polymorphisms in drug-resistant epilepsy in a north Indian population. *Indian Journal of Human Genetics*.

[10] Alekov A. K., Rahman M. M., Mitrovic N., Lerche F., Lerche H. (2000). A sodium channel mutation causing epilepsy in man exhibits subtle defects in fast inactivation and activation in vitro. *The Journal of Physiology*.

[11] Vilin P. C. (2001). Slow inactivation in voltage-gated sodium channels. *Cell Biochemistry and Biophysics*.

[12] Rogawski M. A., Löscher W. (2004). The neurobiology of antiepileptic drugs. *Nature Reviews Neuroscience*.

[13] Xie X., Dale T. J., John V. H., Cater H. L., Peakman T. C., Clare J. J. (2001). Electrophysiological and pharmacological properties of the human brain type IIA Na+ channel expressed in a stable mammalian cell line. *Pflügers Archiv*.

[14] Lakhan R., Kumari R., Misra U. K. (2009). Differential role of sodium channelsSCN1AandSCN2Agene polymorphisms with epilepsy and multiple drug resistance in the north Indian population. *British Journal of Clinical Pharmacology*.

[15] Meisler M. H. (2005). Sodium channel mutations in epilepsy and other neurological disorders. *Journal of Clinical Investigation*.

[16] Löscher W., Klotz U., Schmidt F., Schmidt D. (2009). The clinical impact of pharmacogenetics on the treatment of epilepsy. *Epilepsia*.

[17] Zhou B. T., Zhou Q. H., Yin J. Y. (2012). Effects of SCN1A and GABA receptor genetic polymorphisms on carbamazepine tolerability and efficacy in Chinese patients with partial seizures: 2‐year longitudinal clinical follow‐up. *CNS Neuroscience & Therapeutics*.

[18] Haerian B. S., Baum L., Kwan P., Tan H. J., Mohamed A. A., Mohamed Z. (2013). SCN1A, SCN2A and SCN3A gene polymorphisms and responsiveness to antiepileptic drugs: a multicenter cohort study and meta-analysis. *Pharmacogenomics*.

[19] Abe T., Seo T., Ishitsu T., Nakagawa T., Nakagawa M., Nakagawa K. (2008). Association betweenSCN1Apolymorphism and carbamazepine-resistant epilepsy. *British Journal of Clinical Pharmacology*.

[20] Tate S. K., Depondt C., Sisodiya S. M. (2005). Genetic predictors of the maximum doses patients receive during clinical use of the anti-epileptic drugs carbamazepine and phenytoin. *Proceedings of the National Academy of Sciences*.

[21] Tate S. K., Singh R., Hung C.-C. (2006). A common polymorphism in the SCN1A gene associates with phenytoin serum levels at maintenance dose. *Pharmacogenetics and Genomics*.

[22] Zimprich F., Stogmann E., Bonelli S. (2008). A functional polymorphism in the SCN1A gene is not associated with carbamazepine dosages in Austrian patients with epilepsy. *Epilepsia*.

[23] Sambrook J., Maniatis T. (1989). *Molecular Cloning: A Laboratory Manual*.

[24] Morimoto M., Mazaki E., Nishimura A. (2006). SCN1A mutation mosaicism in a family with severe myoclonic epilepsy in infancy. *Epilepsia*.

[25] Claes L., Del-Favero J., Ceulemans B., Lagae L., Van Broeckhoven C., De Jonghe P. (2001). De novo mutations in the sodium-channel gene SCN1A cause severe myoclonic epilepsy of infancy. *The American Journal of Human Genetics*.

[26] Chou I.-C., Peng C.-T., Tsai F.-J., Huang C.-C., Tsai Y.-R., Tsai C.-H. (2003). The lack of association between febrile convulsions and polymorphisms in SCN1A. *Epilepsy Research*.

[27] Hamdy S. I., Hiratsuka M., Narahara K. (2003). Genotype and allele frequencies ofTPMT,NAT2,GST,SULT1A1andMDR-1in the Egyptian population. *British Journal of Clinical Pharmacology*.

[28] Wirrell E. C. (2013). Predicting pharmacoresistance in pediatric epilepsy. *Epilepsia*.

[29] Depondt C. (2006). The potential of pharmacogenetics in the treatment of epilepsy. *European Journal of Paediatric Neurology*.

[30] Manna I., Gambardella A., Bianchi A. (2011). A functional polymorphism in the SCN1A gene does not influence antiepileptic drug responsiveness in Italian patients with focal epilepsy. *Epilepsia*.

[31] Szoeke C. E., Newton M., Wood J. M. (2006). Update on pharmacogenetics in epilepsy: a brief review. *The Lancet Neurology*.

[32] Shi L., Zhu M., Li H. (2019). SCN1A and SCN2A polymorphisms are associated with response to valproic acid in Chinese epilepsy patients. *European Journal of Clinical Pharmacology*.

[33] Li X., Zhang J., Wu X. (2016). Polymorphisms of ABAT, SCN2A and ALDH5A1 may affect valproic acid responses in the treatment of epilepsy in Chinese. *Pharmacogenomics*.

[34] Sills G., Mohanraj R., Butler E. (2004). A single-nucleotide polymorphism in the scn2a gene is associated with uncontrolled epilepsy: 2.102. *Epilepsia*.

